# Kyste épidermoïde géant de la fesse

**DOI:** 10.11604/pamj.2014.18.286.5031

**Published:** 2014-08-12

**Authors:** Ismail Hmouri, Mohamed Saleh Berrada

**Affiliations:** 1 CHU Avicenne, Rabat, Maroc

**Keywords:** Kyste épidermoïde, fesse, masse, tumeur, epidermoid cyst, buttock, mass, tumor

## Image en medicine

Il s'agit d'un jeune homme de 22ans, sans antécédents pathologiques notables, consulte pour une masse indolore au niveau de la région fessière augmentant insidieusement de volume, mobile par rapport aux deux plans, sans signes inflammatoires en regard (A). Un bilan radiologique a mis en évidence une tumeur kystique d'allure bénigne mesurant 18cm de grand axe (B). Le patient a bénéficié d'une résection chirurgicale en bloc de la tumeur (C). Un examen anatomopathologique de la pièce opératoire a été réalisé objectivant un kyste épidermoïde géant. Les kystes épidermoïdes sont les kystes les plus communs de la peau, ils sont généralement petits et de croissance lente et atteignent rarement plus de 05cm de diamètre.

**Figure 1:**
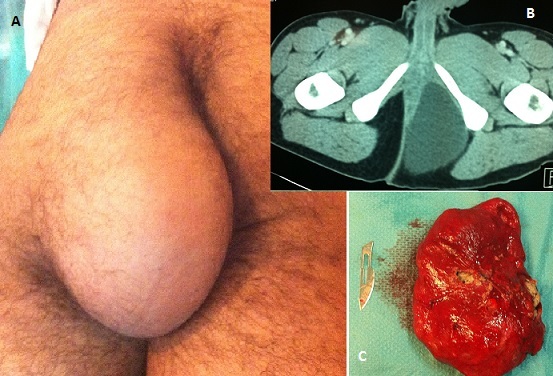
Image clinique montrant la masse au niveau de la fesse gauche

